# Early-Onset TRNT1-Related SIFD Syndrome with an Additional Monoallelic C7 Variant: A Pediatric Case Report

**DOI:** 10.3390/diagnostics16162633

**Published:** 2026-08-19

**Authors:** Iulius Juganaru, Adriana Cojocaru, Andreea Grigorita, Niculina Mang, Andrei Paun, Diana-Georgiana Basaca, Laura Octavia Grigorita, Lucian Ioan Cristun, Otilia Marginean

**Affiliations:** 1 Department XI Pediatrics, Discipline I Pediatrics, ‘Victor Babeș’ University of Medicine and Pharmacy Timișoara, 300041 Timișoara, Romania; juganaru.iulius@umft.ro (I.J.); andrei.paun@umft.ro (A.P.); diana.basaca@umft.ro (D.-G.B.); marginean.otilia@umft.ro (O.M.); 2 1st Department of Pediatrics, Children’s Emergency Hospital ‘Louis Țurcanu’, 300011 Timișoara, Romania; 3 Research Center for Disturbance of Growth and Development in Children BELIEVE, ‘Victor Babeș’ University of Medicine and Pharmacy Timișoara, 300041 Timișoara, Romania; 4 Department of Neurosciences, ‘Victor Babeș’ University of Medicine and Pharmacy Timișoara, 2nd Eftimie Murgu Square, 300041 Timișoara, Romania; adriana.cojocaru@umft.ro; 5 Master’s Program in Applied Auxology in Child and Adolescent Growth and Development, ‘Victor Babeș’ University of Medicine and Pharmacy Timișoara, 300041 Timișoara, Romania; 6 Department I—Discipline of Anatomy and Embryology, Faculty of Medicine, ‘Victor Babeș’ University of Medicine and Pharmacy Timișoara, 2nd Eftimie Murgu Square, 300041 Timișoara, Romania; grigorita.laura@umft.ro; 7 Ph.D. School Department, ‘Victor Babeș’ University of Medicine and Pharmacy Timișoara, 300041 Timișoara, Romania; lucian.cristun@umft.ro

**Keywords:** SIFD, recurrent fever, TRNT1, complement component 7

## Abstract

**Background and Clinical Significance**: Sideroblastic anemia with immunodeficiency, fevers, and developmental delay (SIFD) syndrome is a rare autosomal recessive disorder caused by biallelic variants in the *TRNT1* gene, which encodes tRNA nucleotidyltransferase 1. The disease typically presents early in life with microcytic anemia, recurrent febrile episodes, developmental delay, and immune dysfunction. Because of its rarity and variable clinical expression, diagnosis may be challenging, and broad genetic testing can identify additional variants whose clinical significance requires careful interpretation. **Case Presentation**: We report a 4-month-old male infant with recurrent febrile episodes, severe recurrent microcytic anemia, recurrent infections, mild psychomotor developmental delay, and multisystem involvement. Genetic analysis identified two heterozygous *TRNT1* variants inherited from different parents and confirmed to be in trans: a maternally inherited pathogenic frameshift variant, c.428_431del, p.(Asp143Glyfs12), and a paternally inherited missense variant, c.1246A>G, p.(Lys416Glu), initially classified as a variant of uncertain significance and subsequently interpreted as likely pathogenic in the context of the clinical phenotype and segregation findings. These findings supported the diagnosis of TRNT1-related SIFD syndrome. An additional heterozygous pathogenic C7 variant, c.633_643del, p.(Ser212Hisfs4), was identified. No second pathogenic C7 allele was detected, and functional complement testing was not performed; therefore, complement component 7 deficiency could not be established. **Conclusions**: This case highlights the importance of early genetic evaluation in infants with recurrent fever, microcytic anemia, immune abnormalities, and multisystem involvement. It also emphasizes the need for cautious interpretation of additional monoallelic pathogenic variants detected by broad genetic panels when functional confirmation and a compatible inheritance pattern are lacking.

## 1. Introduction and Clinical Significance

Sideroblastic anemia with B-cell immunodeficiency, periodic fevers and developmental delay is a rare autosomal recessive disorder caused by mutations in the TRNT1 gene [1]. The TRNT1 gene encodes a nucleotidyltransferase enzyme essential for tRNA maturation and protein biosynthesis. Biallelic TRNT1 mutations lead to defective tRNA maturation, disrupting both cytoplasmic and mitochondrial protein synthesis [2]. Additionally, the defect in mitochondrial tRNA processing disrupts heme biosynthesis, leading to ineffective erythropoiesis and accumulation of sideroblasts, which are hallmarks of the sideroblastic anemia observed in SIFD syndrome [3]. The defective mitochondrial function also contributes to immune dysregulation, resulting in B-cell immunodeficiency and recurrent infections [4]. First described in 2013, SIFD syndrome is characterized by a combination of hematological, immunological and neurological abnormalities, including sideroblastic anemia, recurrent febrile episodes, immune dysfunction and developmental delay. Additional features such as sensorineural hearing loss, cardiac involvement, myopathy and retinal abnormalities have been reported, highlighting the multisystemic nature of the disease [5]. Due to its variable expressivity and severity, early diagnosis and multidisciplinary management are crucial.

Broad genetic panels used in the evaluation of suspected immunodeficiency may identify additional variants, whose clinical significance requires careful interpretation. Complement component 7 is involved in the terminal complement pathway, and biallelic pathogenic variants in C7 are associated with autosomal recessive complement deficiency and increased susceptibility to invasive Neisseria infections [6,7,8].

In this case, we present a pediatric patient with early-onset TRNT1-related SIDF syndrome and an additional monoallelic pathogenic C7 variant identified through broad genetic testing.

## 2. Case Presentation

A 4-month-old male infant was admitted to the Pediatric Section III of the Emergency Hospital for Children “Louis Țurcanu” in Timișoara, Romania, with a four-day history of dry cough, nasal obstruction, bilateral conjunctival secretions and a two-day history of fever (maximum recorded intrarectal temperature: 39.6 degrees Celsius). The infant received antipyretic and nonsteroidal anti-inflammatory drug therapy at home without improvement.

### 2.1. Medical History

**Figure 1:**
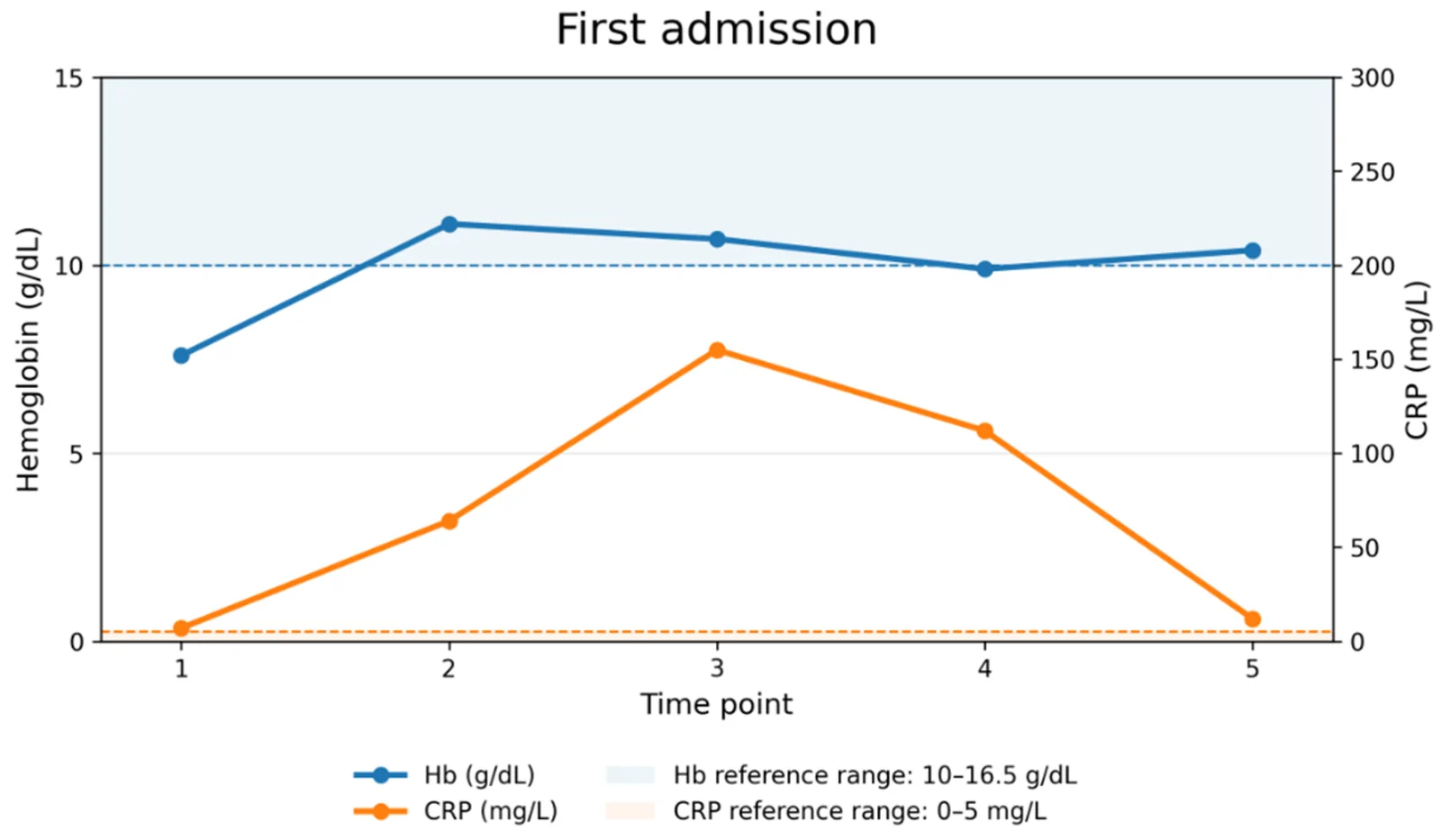
Hemoglobin (Hb) and C–reactive protein (CRP) levels recorded at five time points during the first admission. Hemoglobin is shown on the left *y*–axis and CRP on the right *y*-axis.

**Figure 2:**
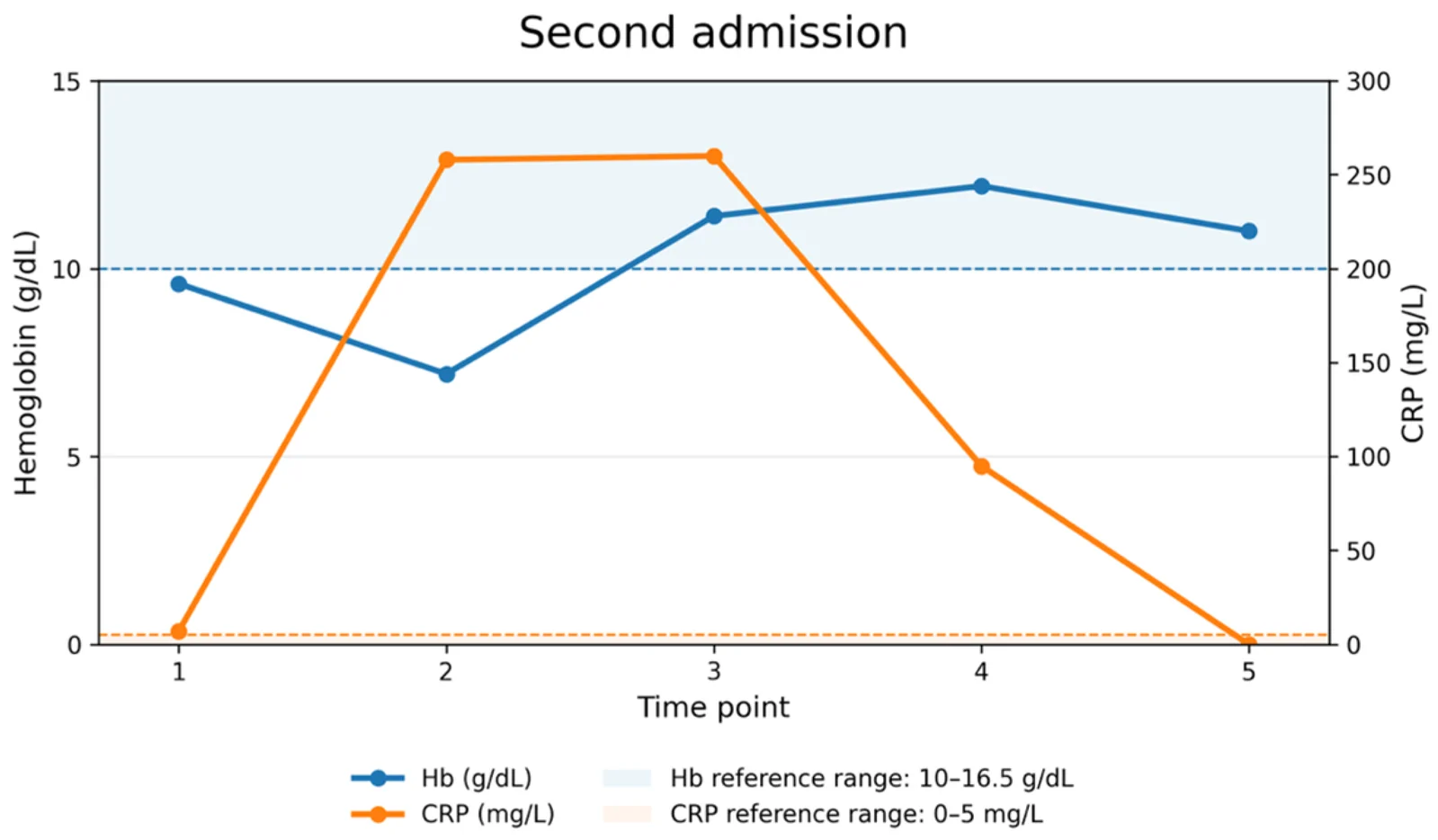
Hemoglobin (Hb) and C–reactive protein (CRP) levels recorded at five time points during the second admission. Hemoglobin is shown on the left *y*–axis and CRP on the right *y*–axis.

**Figure 3:**
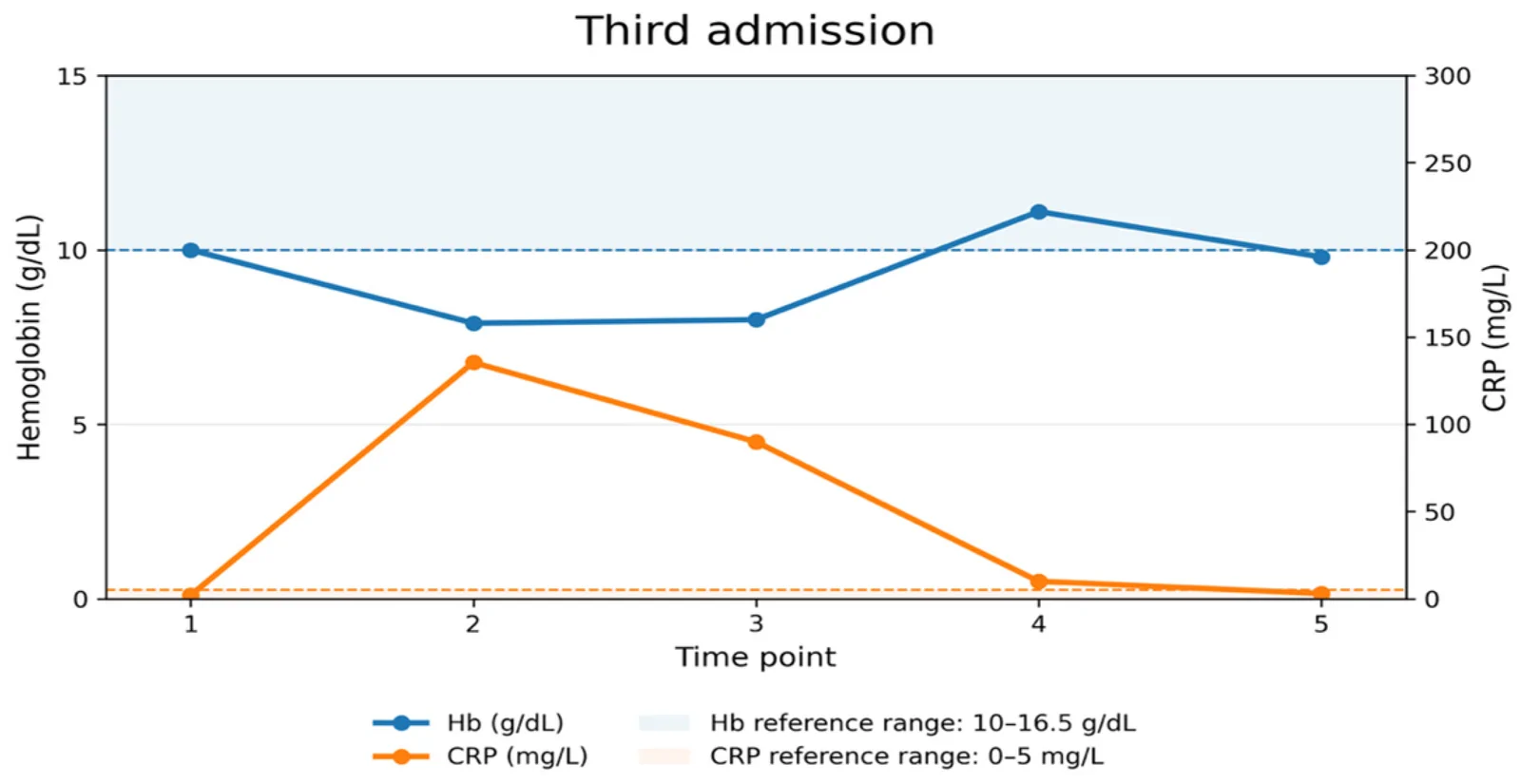
Hemoglobin (Hb) and C–reactive protein (CRP) levels recorded at five time points during the third admission. Hemoglobin is shown on the left *y*-axis and CRP on the right *y*–axis.

- Neonatal period: The patient was born to non-consanguineous parents after a monitored pregnancy, being the first pregnancy and first birth. During gestational week 6, the mother experienced vaginal bleeding, and she received progesterone therapy for it. In the sixth month of pregnancy, she was diagnosed with SARS-CoV-2 infection. The infant was delivered healthy, but with distinctive facial features, via cesarean section at 40 weeks of gestation, with a birth weight of 2710 g, a length of 48 cm and Apgar scores of 8 at 1 min and 9 at 5 min. The family medical history includes a maternal grandmother and maternal uncles with thrombophilia, presenting with deep vein thrombosis and pulmonary embolism.
- Hospitalization at 29 days: The patient was admitted for fever (38.4 degrees Celsius) with elevated inflammatory markers (C-reactive protein: 154.95 mg/L, procalcitonin: 2.6 ng/mL) and hypochromic microcytic anemia (Hb = 7.6 g/dL) (Figure 1). At this age, he had a weight of 3000 g and a length of 52 cm. He was treated with dual antibiotic therapy for eight days and required an isogroup isoRh red blood cell transfusion, with clinical improvement after five days. Genetic evaluation due to dysmorphic features (microcephaly, micrognathia, hypertelorism, low-set ears, and mongoloid palpebral fissures, along with congenital clubfoot—Talus valgus—of the right foot) revealed a normal 46, XY karyotype.
- Hospitalization at 2 months: The patient was rehospitalized for fever without initial symptoms, later developing otitis media, diarrhea with blood streaks and phlebitis at the peripheral venous catheter insertion sites in the upper limb. At this age, he had a weight of 3680 g (weight under the second percentile according to CDC growth charts) and a length of 55 cm. Laboratory findings showed elevated C-reactive protein at 258.35 mg/L, anemia Hb = 7.2 g/dL and no bacterial growth in cultures (Figure 2). He was treated with triple antibiotic therapy and required another transfusion. Clinical improvement occurred after 11 days.
- Hospitalization at 3 months: The patient was hospitalized for recurrent fever syndrome, inflammatory syndrome (CRP = 135.52 mg/dL), microcytic normochromic anemia (Hb = 7.9 g/L) and splenomegaly. At this age, he had a weight of 4240 g (weight under the second percentile according to CDC growth charts) and a length of 58 cm. Following blood investigations and specialist consultations, an acute infectious cause was ruled out (Figure 3). The patient received antibiotic therapy with ceftriaxone and gentamicin, together with systemic corticosteroid therapy with methylprednisolone at 2 mg/kg/day. A bone marrow aspiration that was performed from the calcaneal region for a myelogram and Pearl’s staining showed sideromacrophages (+) but no sideroblasts. Immunologic workup did not support primary immunodeficiency, prompting further investigations for autoinflammatory syndromes. Clinical improvement was observed over the following 14 days.

**Figure 4:**
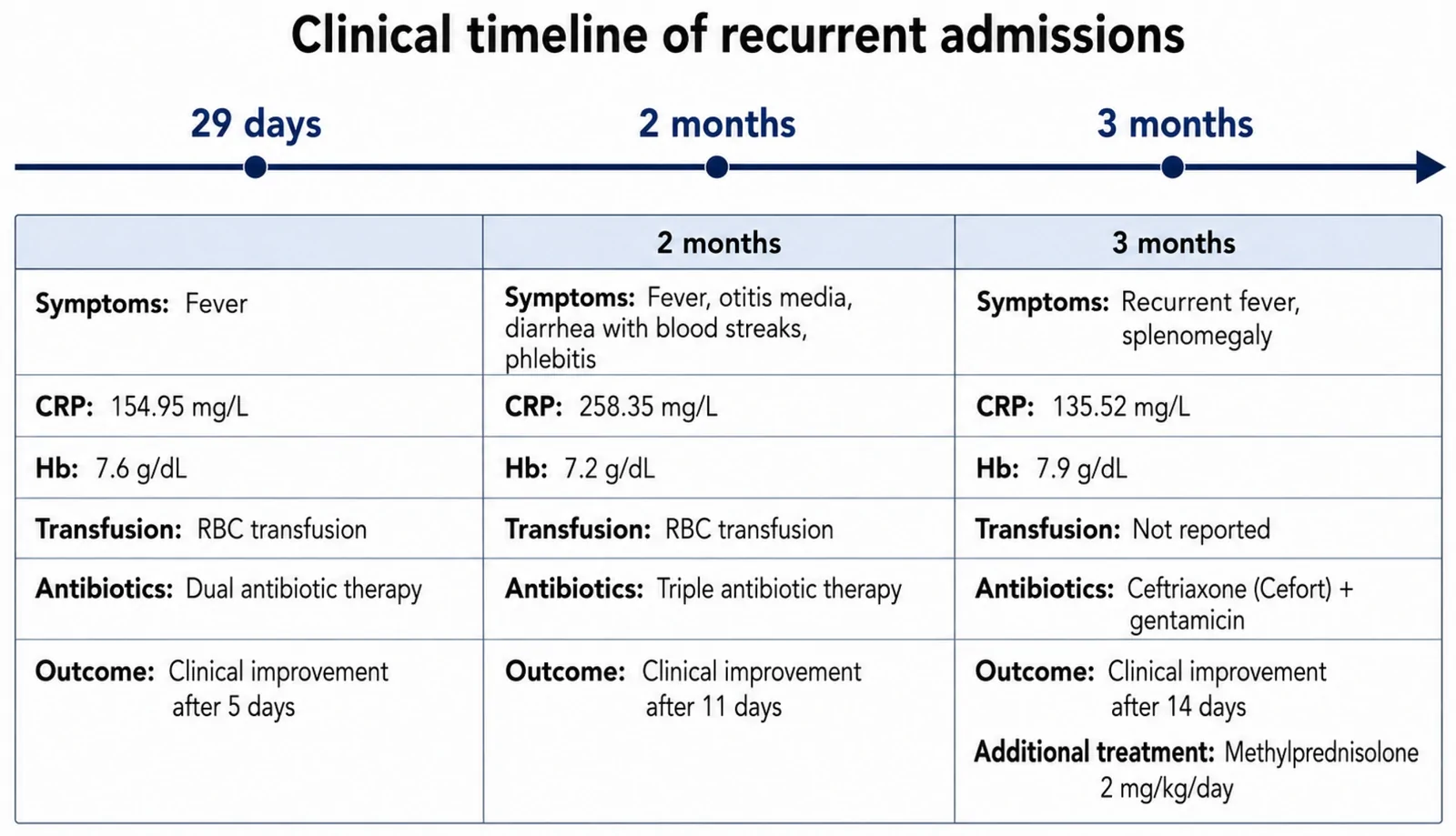
Clinical timeline of recurrent admissions before the current hospitalization summarizing the age at admission, major symptoms, CRP and hemoglobin values, transfusions, antibiotic therapy, and clinical outcome.

A summary of the recurrent admissions, including major symptoms, CRP and hemoglobin values, transfusions, antibiotic therapy, and clinical outcomes is presented in Figure 4.

### 2.2. Signs and Symptoms

**Figure 5:**
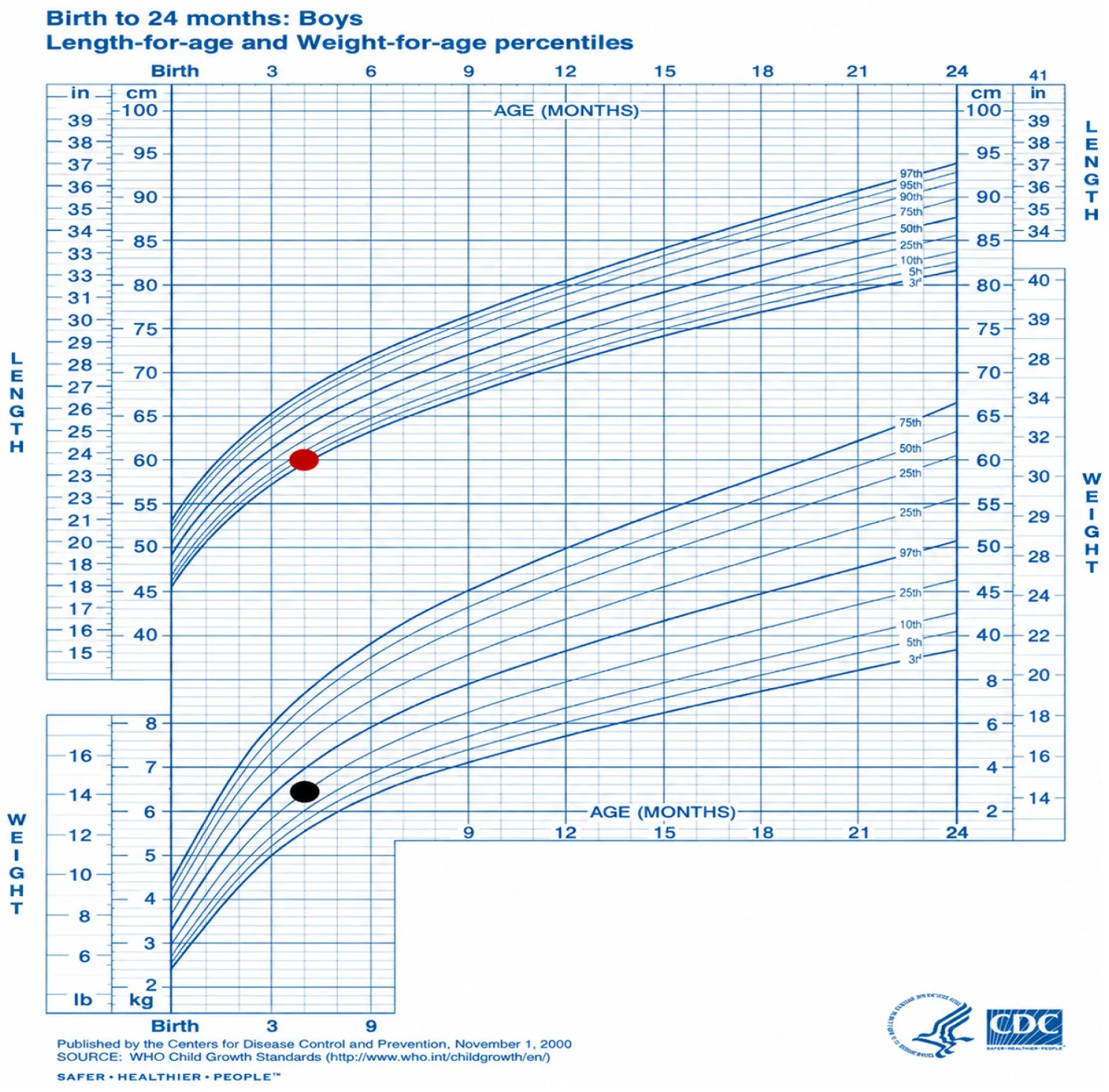
Growth parameters at 4 months: CDC standards vs. patient data.

**Figure 6:**
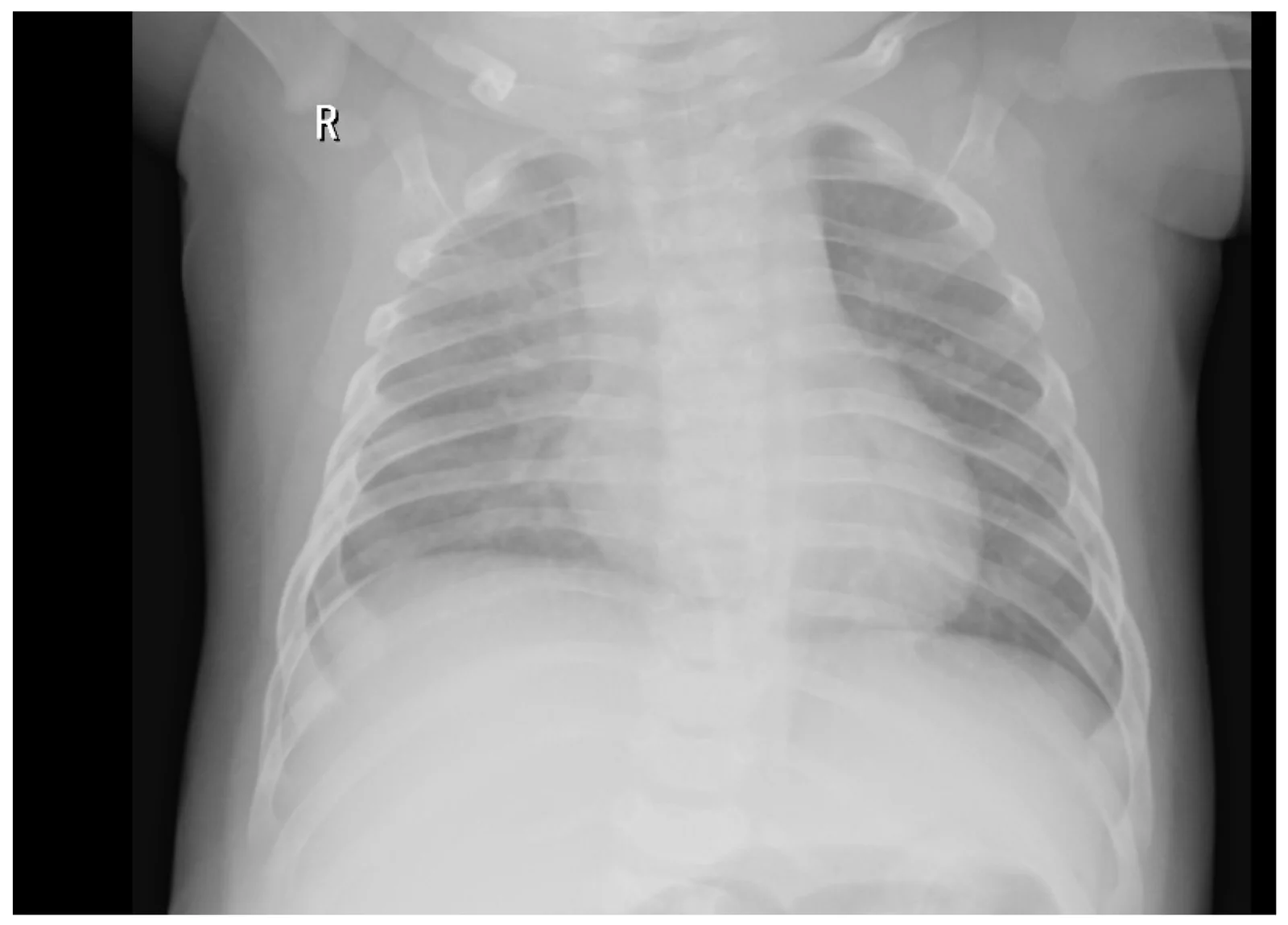
Chest X-ray showing bilateral paramediastinal interstitial infiltrates, more pronounced on the right, without consolidated foci.

At the present admission at 4 months of age, the clinical examination revealed craniofacial dysmorphism with microcephaly, micrognathia, hypertelorism, low-set ears and upslanted palpebral fissures, hyperemic conjunctivae, congested pharynx with candidiasis deposits, symmetrical bilateral vesicular breath sounds, right-sided subcrepitant rales, and rhonchi without respiratory distress. Abdominal examination showed diastasis of the rectus abdominis muscle, a distended abdomen, present bowel transit, hepatomegaly (+2 cm below the right costal margin) and a palpable spleen. Mild psychomotor delay was noted. Auxological parameters: weight 6.300 g, height 60 cm, ponderal index 1.1. His weight of 6.300 g is between percentiles 10 and 25, the patient being positioned slightly below average in weight, while his height of 60 cm is on the second percentile, the patient being positioned below average in length (Figure 5). The improvement in weight percentile compared with previous admissions may reflect catch-up growth during a period of clinical stabilization; however, detailed nutritional intake data were not available. Although this improvement followed red blood cell transfusion and treatment of the recurrent inflammatory episodes, a direct causal relationship with transfusion therapy cannot be established. A chest X-ray performed in the emergency department showed bilateral paramediastinal interstitial infiltrates, more pronounced on the right, without consolidated foci or thymic hypertrophy (Figure 6). Based on the clinical examination and chest X-ray, a presumptive diagnosis of interstitial pneumonia was made.

### 2.3. Biological Investigations

At the time of admission to the pediatrics department, the patient presented with elevated ferritin levels (475 ng/mL), microcytic hypochromic anemia, mild monocytosis, negative procalcitonin and mildly elevated C-reactive protein (18.6 mg/L). The anemia was persistently microcytic, with mean corpuscular volume values remaining below the age-adjusted range throughout hospitalization, from 72.1 fL (reference range 87–109 fL) on day 1 to 71.7 fL at discharge.

A rapid antigen test for SARS-CoV-2 and Influenza A and B was performed, with negative results. The blood tests revealed decreased serum iron (1.5 umol/L) and reduced IgG levels compared to the age-specific reference range of 2.32–14.11 g/L (patient value: 1.99 g/L). IgA was <0.20 g/L (reference range 0–0.83 g/L) and IgM was <0.13 g/L (reference range 0–1.45 g/L). Detailed lymphocyte subset analysis, including B-cell quantification, was not available. No pathogenic bacteria were detected in the blood cultures, throat swab, nasal swab or conjunctival secretion culture. The urinalysis showed no abnormalities. The abdominal ultrasound revealed splenomegaly, with a splenic long axis of 8.5 cm, with no other abnormalities. The patient was also tested for occult bleeding, with a positive result. Over the following days, the patient experienced febrile spikes and daily episodes of semi-formed and diarrheic stools. Stool tests for antigens (Rotavirus, Norovirus, Adenovirus and Campylobacter) and stool culture were performed, all with negative results despite the increase in inflammatory markers, with C-reactive protein reaching a maximum of 118.96 mg/L and procalcitonin 2.61 ng/L.

The evolution of the main inflammatory and hematological parameters, including CRP, procalcitonin, ferritin, white blood cells, red blood cells and hemoglobin, is summarized in Table 1.

### 2.4. Other Examinations

Genetic investigations were initiated, including an immunodeficiency gene panel: sequence analysis and deletion/duplication testing of the 452 genes are listed in the Genes Analyzed Section (Invitae Primary Immunodeficiency Panel, Add-On Primary Ciliary Dyskinesia and Cystic Fibrosis Genes, and Add-On Cancer Predisposition Genes) (Table 2).

Genetic evaluation identified two heterozygous TRNT1 variants inherited from different parents: a maternally inherited pathogenic frameshift variant, c.428_431del, p. (Asp143Glyfs*12), and a paternally inherited missense variant, c.1246A>G, p. (Lys416Glu), initially classified as a variant of uncertain significance (VUS) in the 2022 Invitae report. Subsequent segregation analysis confirmed that the two variants were inherited from different parents and were therefore in trans. In the subsequent clinical genetic evaluation, the c.1246A>G variant was interpreted as likely pathogenic in the context of the patient’s compatible phenotype and segregation findings, supporting the diagnosis of SIFD syndrome. Current variant-level evidence further supports the clinical relevance of c.1246A>G, p. (Lys416Glu). ClinVar currently classifies this variant as pathogenic/likely pathogenic, with multiple submitters and no conflicting interpretations for SIFD [9]. ACMG/AMP-based assessments reported for this variant include PM3_Strong, PM2_Supporting and PP1, reflecting its occurrence in trans with pathogenic TRNT1 variants, its rarity in population databases and segregation with disease [9]. The Lys416 residue is evolutionarily conserved, and the variant has been identified in several individuals with TRNT1-related disease, frequently in compound heterozygosity with another pathogenic or likely pathogenic TRNT1 variant [1,9,10,11,12]. In silico evidence is not fully concordant; therefore, computational predictors were considered supportive but not independently decisive.

An additional monoallelic pathogenic C7 variant, c.633_643 del, p. (Ser212Hisfs*4), was reported. As no second pathogenic C7 allele was identified, this finding was not considered diagnostic of complement component 7 deficiency. Functional complement assay (CH50 and AH50) and serum C7 protein levels were not performed.

More extensive functional characterization of the SIFD phenotype, including detailed B-cell phenotyping and immunoglobulin subclass analysis, was not available. Specific mitochondrial functional studies were also not performed.

Both parents were clinically healthy, with no manifestations suggestive of SIFD. The patient had no siblings, and no other family members were reported to have a similar clinical phenotype.

Additionally, a pediatric neurology consultation was conducted, and an EEG during natural sleep was performed, revealing a medium-voltage delta background rhythm without pathological graphoelements. A mild delay in psychomotor development was also noted.

From an ENT perspective, acute mucopurulent rhinitis is noted, with no pathological changes at the otic level. Ophthalmological evaluation reveals acute conjunctivitis and lacrimal duct stenosis.

## 3. Discussion

We present a 4-month-old boy with a particular phenotype characterized by microcephaly, micrognathia, hypertelorism, low-set ears, upslanted palpebral fissures, and SIFD syndrome, who underwent multiple hospitalizations for recurrent infections with an unidentified pathogen, severe recurrent microcytic anemia and mild psychomotor developmental delay. Genetic analysis identified compound heterozygous TRNT1 variants inherited from different parents: a maternally inherited pathogenic frameshift variant, c.428_431del, p. (Asp143Glyfs*12), and a paternally inherited likely pathogenic missense variant, c.1246A>G, p.(Lys416Glu), supporting the diagnosis of SIFD syndrome [9]. Additionally, a monoallelic pathogenic C7 variant, c.633_643del., p. (Ser212Hisfs*4), was reported as an incidental genetic finding.

In a systematic review conducted in 2022, Maccora et al. (2023) [2] analyzed 58 cases of sideroblastic anemia with B-cell immunodeficiency, periodic fever and developmental delay (SIFD) syndrome. The study found that the median age of symptom onset was 4 months, with common manifestations including microcytic or sideroblastic anemia (93.1%), recurrent fevers (89.6%), neurological impairments (82.7%) and humoral immunodeficiency (82.7%). Other frequent features were gastrointestinal symptoms (63.7%), ocular conditions like cataracts and retinitis pigmentosa (46.5%), failure to thrive (45.8%), mucocutaneous issues (50%), and sensorineural deafness (32.7%). In our case, up until diagnosis, the patient exhibited symptoms that are most frequently reported in the literature. This suggests that despite the identification of 41 newly reported genetic mutations among the 58 studied patients, key clinical manifestations such as microcytic/sideroblastic anemia, recurrent fever, neurological delay and immunological abnormalities are present in over 80% of cases.

Despite markedly elevated inflammatory markers, in our case, repeated cultures remained negative. In the context of TRNT1-related SIFD, some episodes may have represented sterile autoinflammatory flares. However, partially treated bacterial infections or unidentified viral infections cannot be excluded. Thus, both infectious and autoinflammatory mechanisms may have contributed to the recurrent febrile episodes.

The differential diagnosis included other disorders presenting in infancy with severe anemia and multisystem involvement. Diamond–Blackfan anemia was considered because of the early-onset transfusion-dependent anemia; however, this disorder is typically characterized by pure red-cell aplasia with normochromic, usually macrocytic anemia, whereas our patient had recurrent microcytic anemia and a broader inflammatory/immunological phenotype. Pearson syndrome was also considered because it may present in infancy with severe transfusion-dependent anemia and multisystem involvement; however, the overall clinical and genetic findings supported TRNT1-related SIFD as the more consistent diagnosis. Severe congenital neutropenia can present with early recurrent infections and fever, but persistent severe neutropenia was not a feature of our patient. Finally, other autoinflammatory syndromes were considered because of the recurrent fever and marked inflammatory response; however, the combination of hematologic, immunological, clinical, and genetic findings was most consistent with TRNT1-related SIFD.

Mutations in TRNT1 impair the enzyme’s ability to add the CCA sequence to the 3′ end of tRNA molecules, a critical step in tRNA maturation [10]. This deficiency disrupts mitochondrial protein synthesis, leading to compromised mitochondrial function [11]. Studies show that the interplay between TRNT1 mutations, mitochondrial dysfunction and viral infections can lead to significant neurological complications in pediatric patients with SIFD syndrome, suggesting that the viral illness may act as a trigger for neurological deterioration. Khojah et al. (2024) [12] report a pediatric case of stroke-like episodes with mitochondrial encephalomyopathy secondary to SARS-CoV-2 infection in a patient with TRNT1 mutations, highlighting the necessity for heightened vigilance in differentiating between psychomotor developmental delay associated with SIFD syndrome and the variable possible complications of a viral infection, which may act as a trigger for neurological manifestations.

However, since the first report of SIFD, researchers have discovered other patients with disease-causing mutations in TRNT1, some of whom exhibit symptoms that differ from the original description. Because TRNT1 is encoded in the nucleus but functions in both the cytoplasm and mitochondria, certain mutations may interfere with its proper folding once inside the mitochondria. This could explain the diverse range of phenotypes observed, just as Mendoca et al. (2021) [6] describe a case of SIFD syndrome without the classic signs of the disease, such as sideroblastic anemia and neurological delay, but with recurrent fever and a skin presentation featuring erythema nodosum. Bardou et al. (2021) [13] describe a case of SIFD syndrome with erythematous papular and nodular skin lesions localized on the limbs and trunk; histopathological examination classified these lesions as a neutrophilic dermal infiltrate. In our case, the patient presented with oral candidiasis, which is a common manifestation due to immune system dysfunction, but mucosal involvement in SIFD syndrome can manifest in various ways, including oral ulcers, gingival inflammation and other mucosal erosions [14].

Although sideroblastic anemia is a characteristic feature of SIFD syndrome [2], bone marrow examination in our patient showed sideromachrophages but no identifiable sideroblasts. A bone marrow aspiration was performed at 3 months of age, after two previous red blood cell transfusions. These transfusions may have influenced erythropoietic activity and complicated the interpretation of the marrow findings, although their contribution to the absence of sideroblasts cannot be established with certainty. A repeat bone marrow examination was not performed during the reported follow-up. Therefore, sideroblastic anemia was not morphologically confirmed in this patient, whose hematologic presentation was characterized by severe recurrent microcytic anemia.

In our patient, an additional monoallelic pathogenic C7 variant was identified and was considered an incidental genetic finding rather than evidence of a second confirmed immunodeficiency. C7 is a critical component of the complement system, which plays a pivotal role in the immune response, particularly in defending against bacterial infections. However, complement component 7 deficiency is an autosomal recessive condition and the identification of a single pathogenic allele is insufficient to establish the diagnosis [7,8,15]. No second pathogenic C7 allele or functional complement investigations, including CH50, AH50 or serum C7 measurement, were available. Therefore, the clinical contribution of the monoallelic C7 finding to the recurrent infectious or inflammatory manifestations could not be established. As the monoallelic C7 finding was not considered diagnostic of complement deficiency, no specific meningococcal vaccination strategy or long-term antibiotic prophylaxis was initiated on this basis.

As no standardized treatment guidelines currently exist for this condition, we aimed to summarize the therapeutic approaches reported in the literature. While supportive care—including transfusions, immunoglobulin replacement, antibiotics, and electrolyte supplementation—was commonly used, some patients received disease-modifying treatments. These included hematopoietic stem cell transplantation, which showed variable outcomes, and anti-TNFα agents such as Etanercept and Infliximab, which were associated with clinical improvement in several cases [2]. Treatment with a TNF inhibitor led to reduced inflammation, decreased transfusion requirements, and improved growth [16,17]. Other therapies, including Anakinra, corticosteroids, colchicine, methotrexate, and azathioprine, were used with mixed results, often based on individual symptoms and physician experience.

## 4. Conclusions

This case highlights the diagnostic complexity and clinical significance of TRNT1-related SIFD syndrome. The identification of SIFD syndrome due to compound heterozygous TRNT1 variants in a 4-month-old child, along with an incidental monoallelic pathogenic C7 variant, underscores the importance of comprehensive genetic and immunologic evaluation in infants presenting with multisystem involvement. While the clinical phenotype was primarily driven by SIFD syndrome, the clinical significance of the additional C7 finding could not be established in the absence of a second pathogenic allele or functional complement testing. This case illustrates the value of integrative genomic diagnostics and proactive surveillance in guiding management and improving outcomes in children with complex immunogenetic disorders.

Several limitations of this case should be acknowledged. More extensive functional immunological characterization, including detailed B-cell phenotyping and immunoglobulin subclass analysis, was not available. Functional complement assays (CH50 and AH 50) and serum C7 protein levels were not performed. Specific mitochondrial functional studies were also not available. These limitations restrict the functional characterization of the reported genetic findings and should be considered when interpreting the present case.

## Figures and Tables

**Table 1:** Trends in inflammatory and hematological parameters during hospitalization.

	D1	D3	D4	D6	D9	D11	D13	D15	D17	Reference Values
CRP	18.06	51.48	-	15.51	10.06	90.43	118.96	7.75	0.86	0–5 mg/L
RBC	3.55	-	3.35	3.69	3.74	3.52	3.43	-	4.06	3.5–5.2 × 10^6^/µL
HGB	8.3	-	7.6	8.2	8.5	7.8	7.6	-	8.9	10–16.5 g/dL
Ferritin	475	-	-	305	-	-	-	490	-	20–200 ng/mL
Procalcitonin	0.250	0.413	-	-	-	2.61	2.57	0.739	0.117	0–0.5 ng/mL
WBC	8.64	-	6.69	5.38	10.41	-	10.73	-	10.17	5–17 × 10^3^/µL

**Abbreviations:** CRP, C-reactive protein; RBC, red blood cells; HGB, hemoglobin; WBC, white blood cells.

**Table 2:** Identified TRNT1 variants in patient.

Gene	Nucleotide Change	Protein Change	Zygosity	Variant Classification	Inheritance	Predicted Impact
TRNT1	c.428_431del	p.Asp143Glyfs*12	Heterozygous	Pathogenic ^1^	Maternal	Frameshift, early stop codon
TRNT1	c.1246A>G	p.Lys416Glu	Heterozygous	VUS in the original 2022 report; currently classified as likely pathogenic ^2^	Paternal	Missense, substitution

^1,2^ One pathogenic variant and one variant initially classified as a variant of uncertain significance (VUS) were identified in TRNT1. Parental testing demonstrated maternal inheritance of c.428_431del and paternal inheritance of c.1246A>G, confirming that the two variants are in trans and establishing compound heterozygosity. The c.1246A>G, p. (Lys416 Glu) variant was subsequently interpreted as likely pathogenic by the clinical geneticist in the context of segregation findings and the compatible clinical phenotype. These findings support the diagnosis of autosomal recessive TRNT1-related SIFD syndrome.

## Data Availability

The data are not publicly available for privacy reasons.

## Abbreviations

The following abbreviations are used in this manuscript:

C7	Complement 7
CRP	C-reactive protein
Hgb	Hemoglobin
MCV	Mean corpuscular volume
RBC	Red blood cells
SIFD	Sideroblastic anemia with immunodeficiency, fevers, and developmental delay
WBC	White blood cells
